# Impact of Dynamic Soil-Structure Interaction on Performance of a Single Span Footbridge with Overhangs Subjected to Mining-Induced Shocks

**DOI:** 10.3390/ma15249084

**Published:** 2022-12-19

**Authors:** Izabela Joanna Drygala, Joanna Maria Dulińska, Nicola Nisticò, Tadeusz Tatara

**Affiliations:** 1 Faculty of Civil Engineering, Cracow University of Technology, 31-155 Cracow, Poland; 2 Department of Structural and Geotechnical Engineering, Sapienza University of Rome, 00184 Rome, Italy

**Keywords:** footbridge, dynamic soil-structure interaction, mining-induced seismicity, dynamic performance of bridges, field test, operational modal analysis, FEM models

## Abstract

The impact of the dynamic soil-structure interaction (DSSI) on the response of a single-span footbridge to mining-induced shocks was assessed. Firstly, the eigen values, modes and damping of the footbridge were evaluated based on in-operation field tests. Then, natural frequencies were determined numerically by a model usually used in static calculations, i.e., a simple supported beam with overhangs. The numerical natural frequencies turned out to be inconsistent with the experimentally determined values. In turn, the model, assuming the overhangs’ ends translationally restrained, gave natural frequency values closer to the experimental ones. However, for the third mode, that is lateral, the frequency error (~26%) can be considered greater than usually accepted values. Hence, the three-dimensional numerical model of the footbridge was tuned by considering the DSSI between the overhangs and the ground, and implementing springs (in three directions) at the overhangs’ ends. To estimate the impact of DSSI on the dynamic performance of the footbridge, time history analyses were carried out for the model with fixed overhang ends and for the model with additional springs. Two different types of mining-induced tremors were used as excitations. Those two tremors (narrow and wide band) induced different dynamic responses in the models with and without the springs. Hence, the impact of the DSSI on the dynamic footbridge performance needs to be considered to predict the effect of mining-induced shocks.

## 1. Introduction

In engineering practice, single-span footbridges with a span of about 30–50 m are often adopted as crossings on both lower-velocity streets and major roadways [1]. The quoted length of such structures is usually the result of meeting the length of the obstacle, the ultimate limit state (ULS) requirements, and the serviceability limit state (SLS), as well as economic issues [2,3]. If the required total span of the pedestrian bridge is longer than 30–50 m alternative solutions include simple-supported beams with overhangs. It is worth noticing that the end steel parts of the girders need protection against the soil induced aggressive environment: the solution, as usually adopted in Poland, can be implemented by embedding those parts into an additional concrete basement. Depending on the weight of the concrete as well as the live load and soil settlement those basements can result in contact or not with the soil. Consequently, the structural system of the deck results in a beam either with four pin supports or with two pin supports and two overhangs. When the second solution is adopted the discussion on considering the dynamic soil-structure interaction (DSSI) is reasonable in the context of response of the structure to mining-induced shocks.

The physical phenomenon of DSSI has been extensively investigated in recent decades. In general, the DSSI could be described as an action in which the soil’s response acts on the structure’s motion and vice versa [4]. Reissner [5] initiated the study on DSSI in 1936 by researching the behavior of circular disks on elastic half-spaces subjected to time-harmonic vertical force. Then, the DSSI issues were explored concerning the seismic assessment of structures [6,7,8]. Authors mainly examined the DSSI effects for stiff, massive, and slightly damped systems [9]. It was also stated that the detrimental effect of SSI on the structural response could affect the seismic demand in terms of the structural load capacity [10,11]. 

The DSSI problems have been discussed and explored in the case of footbridges as well. It was recognized [12,13] that DSSI effects could modify the modal properties of footbridges, so that such investigation could be required for footbridges exposed to Mining-Induced Shocks.

Nowadays, in addition to taking into account natural seismic phenomena, there is an urgent need to protect the existing and designed engineering structures against mining-induced seismic shocks occurring in mining activity regions. The evaluation of risk resulting from mining-triggered events to surface structures has become a task of recent studies. However, most of the papers concern the influences of these tremors on residential buildings [14,15,16,17]. The recognition of mining-related impacts on other engineering structures is still insufficient. In particular, the effects of mining-induced seismicity on footbridges are not well recognized yet.

Mining-triggered tremors exhibit characteristic features that distinguish them from natural earthquakes. The differences relate mainly to the mechanism of shock generation, the wave propagation from the source to the receiver, the range of dominant frequencies, duration, and repeatability of occurrence [18,19,20]. In a typical record of mining-triggered vibrations [21], in close proximity to the source, amplitudes of vertical oscillations are comparable to or even higher than amplitudes of horizontal vibrations. Thus, the surface points undergo complex spatial movements, and all vibrational components must be considered in the analyses concerning mining-induced shocks. The dominant frequency value of typical mining-induced tremors is usually higher than 5 Hz [22]. However, it is also not uncommon that frequency spectra are very scattered, covering all frequencies up to 20 Hz, and not having dominant frequencies [21]: this implies resonance problems with higher frequencies of footbridges. 

The analyses of the dynamic performance of structures under mining-related quakes are similar to those concerning natural earthquakes. However, in the case of the dynamic analysis of footbridges subjected to mining tremors, the comparable size of the three components of kinematic excitation seems to be crucial for the analysis accuracy. Further, numerical models need to be properly tuned to evaluate vertical- as well transversal-induced vibrations [23,24] due to dynamic action such as those induced by pedestrians. 

The main objective of the paper is to assess the impact of the DSSI on the dynamic performance of a concrete–steel composite footbridge with overhangs interacting or not with the soil when subjected to mining-induced shocks. To quantify the impact of the DSSI, the dynamic response levels obtained for two representative mining-induced shocks were investigated by means of FEM analyses, considering, the end part of the overhang either fixed or supported by elastic springs, representative the material properties of soil. Those spring stiffnesses have been tuned fitting the numerical frequencies with those experimentally evaluated through in field test and Operational Modal Analysis (OMA). 

Due to footbridge typology and the nature of the considered excitation (i.e., the mining-induced seismic excitation was investigated), the study presented here can be considered a novelty as far as the authors’ knowledge is concerned.

## 2. Materials and Methods 

The main objective of the research was to show the influence of the DSSI on the seismic assessment of a concrete-steel composite footbridge with overhangs. Firstly, the experimental modal model was completed during field tests. Then, the footbridges’ finite element (FE) model was assembled and validated. The last stage was the seismic assessment of the structure subjected to mining-induced shocks. 

### 2.1. Structural Layout and Material Data of the Footbridge

**Figure 1:**
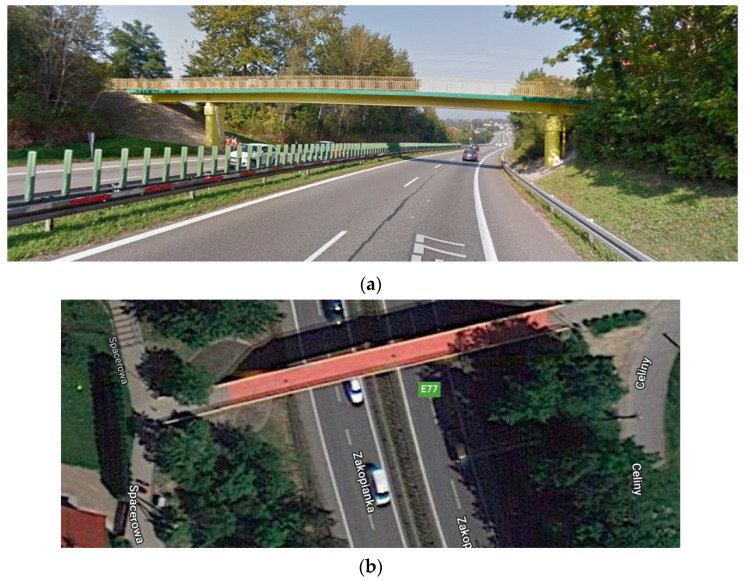
(**a**) General view of the analyzed footbridge, (**b**) location of the structure (source: google.com/maps/place/Celiny,+32-031+Mogilany/@49.9441301,19.891348,86m/data=!3m1!1e3!4m5!3m4!1s0x471667e327866d15:0x2be86a9c42b5bf72!8m2!3d49.9452772!4d19.8960001 (accessed on 12 December 2022)).

**Figure 2:**
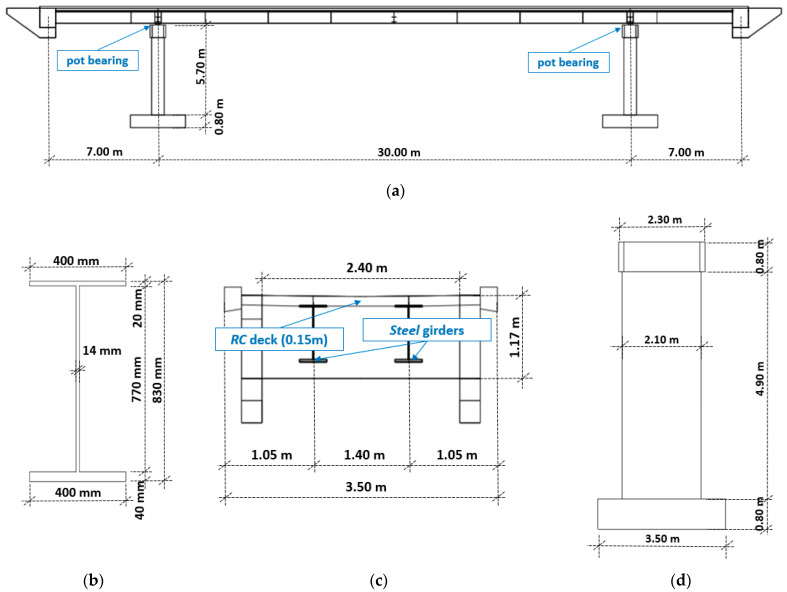
Footbridge geometry: (**a**) side view, (**b**) steel girder cross-section (**c**) deck cross-section (**d**) pier section.

The investigated footbridge is located in Mogilany, Southern Poland, over the national road S7 (Figure 1) [25]: (1) the central span 30 m long (Figure 2a) as well as the two lateral spans (with a global length equal to 14 m), consist of composite steel girders (Figure 2b) connected to a concrete slab ≈15 cm thick (Figure 2c) through 814 ∅ 16 (mm) steel nelson type connectors (h = 100 mm) placed, in the longitudinal direction, with a step of ~200 mm, organized in group of 2 (#114) and 4 (#744); (2) at the end of the lateral spans two concrete blocks: those blocks, with steel beams embedded inside, serve to protect the steel beams against corrosion triggerable by soil environment and as an extreme support for the two lateral overhangs; (3) pot bearings are placed between the deck and the two reinforced concrete pears (Figure 2d). 

Adopted material data and element masses are summarized in Table 1.

**Figure 3:**
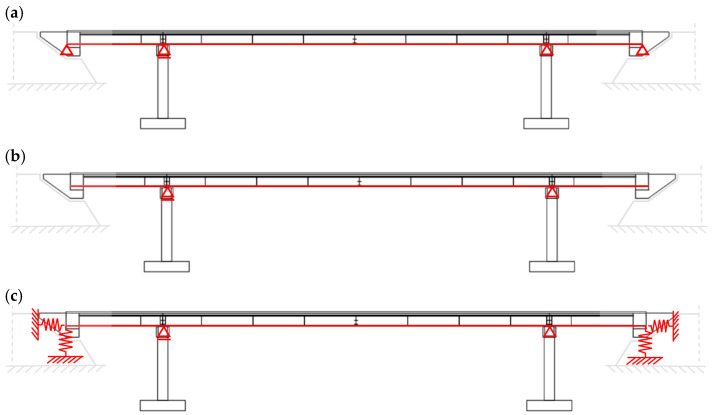
Footbridge structural schematization (red shapes): (**a**) FIX_Ovhg model (**b**) FREE_ Ovhg model, in which overhangs do not interact with the ground (**c**) SPRING_ model with overhangs connected to the ground by springs.

Due to the concrete blocks, it could be argued that the adopted design structural system, in the case of live load, was a beam supported by 4 hinge-like supports, as reported in Figure 3a, but cannot be a priori excluded that the end part of the overhangs is not restrained at all (Figure 3b). Hence, when static loadings were considered, the scheme when overhangs do not interact with the ground was applied as one of the cases for envelopes of internal forces [25].

**Figure 4:**
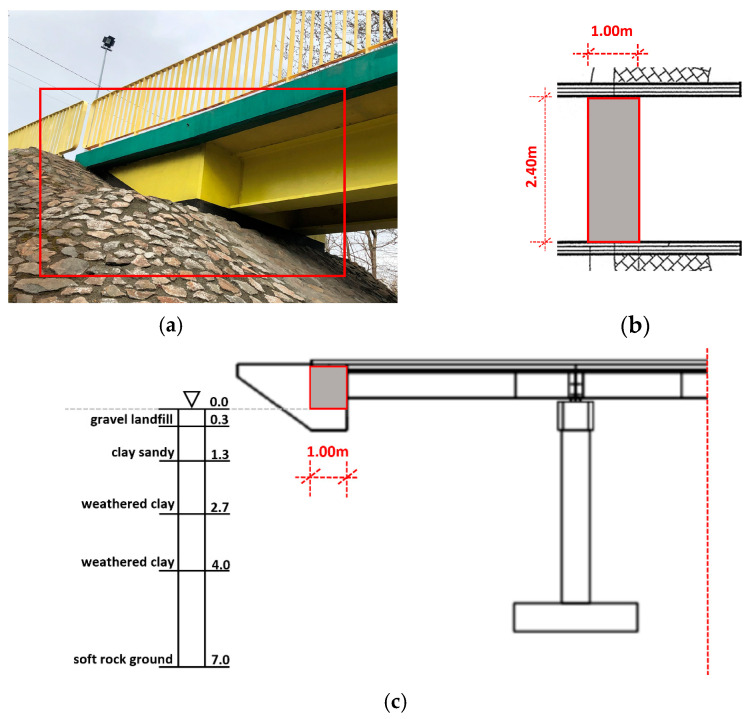
The steel-concrete composite footbridge: side view (**a**) and horizontal projection (**b**) of the overhang with the DSSI zone marked; (**c**) subsoil geotechnical profile from field test.

In light of the above considerations, (1) the end parts of the overhangs (Figure 4a) were investigated in order to define the contact area, which dimensions (Figure 4b), in the horizontal plane, are 1.0 × 2.40 m; (2) data on the subsoil profile, recognized experimentally throughout field tests at the design stage, were acquired and (3) the subsoil profile was defined as reported in Figure 4c: it consists of gravel landfill, sandy clays, weathered clays (with consistency index $I_{L}=0.1$).

### 2.2. Experimental Set-Up 

Clearly, in general, the model could include a system of springs to take into account the interaction between soil and structure. Hence, the discussion on the DSSI effect on the behavior of the footbridge is reasonable and is not questionable in case of exploitation. Consequently, numerical analyses will be presented in the following sections focusing on the effect of mining-induced shocks on the footbridge 3D modeled supposing the 3 assumptions reported in Figure 3. 

**Figure 5:**
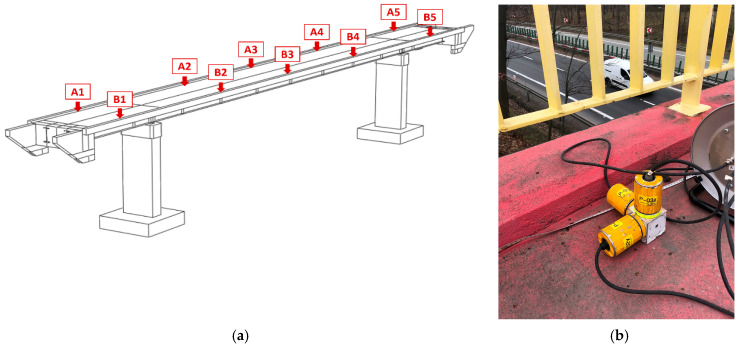
(**a**) Layout of the measurement points located along the footbridge (point A1–A5, B1–B5); (**b**) The A1 measurement point equipped with three piezoelectric high sensitivity (10,000 mV/g) accelerometers.

Adopted spring stiffness, starting from their prediction through literature expressions, was identified through in situ tests that included the installation of a set of accelerometers and signal acquisition that served for the carried out Operational Modal Analysis (OMA) technique [26,27,28,29,30]. The experimental set-up, implemented for the field tests, is shown in Figure 5: (1) all control points were equipped with three piezoelectric high sensitivity (10,000 mV/g) accelerometers 393B12 PCB Piezotronics (wire connected), acting in three directions: (a) the frequency range of accelerometers was from 0.15 to 1000 Hz, (b) data sampling of the signal was 1024 Hz; (2) the measurement points, A1–A5 and B1–B5, were located (Figure 5) on the footbridge slab above each girder.

Those accelerometers were used to acquire the histories consequent to environmental excitations. The modal parameters of the footbridge were estimated based on the in-operation measurements, considering the auto and cross-correlation of signals [27]. The peak picking method [28] was used to estimate the natural frequencies, while the mode associated with each natural frequency was evaluated through the Time Domain Decomposition (TDD) method [30]. To avoid modes correlated with the same pole of system, the version of Modal Assurance Criterion (MAC) called AutoMAC [31] was used, i.e., the set of experimental modes was verified in terms of similarity and orthogonality of eigenvectors. 

The acquired environmental data were the base of the evaluation of the damping properties through the evaluation of logarithmic damping decrements [32] during the free vibrations opportunely selected by the band-pass filter [33]. 

## 3. Mining-Induced Shock Scenario

**Figure 6:**
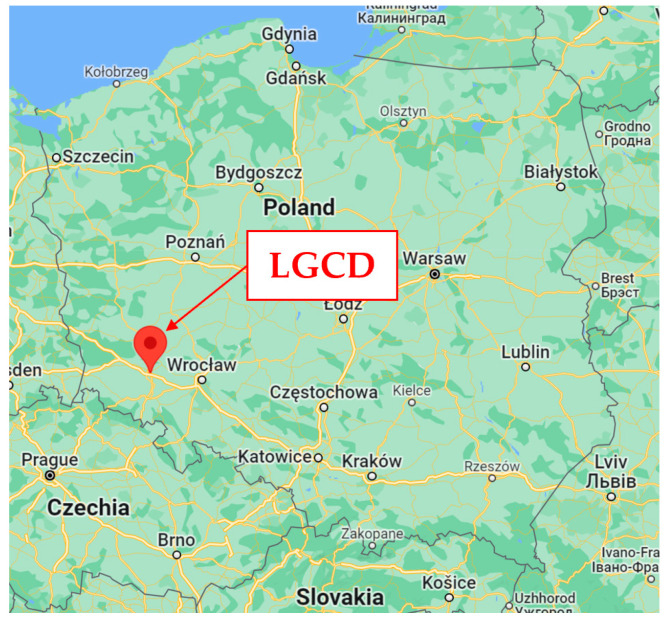
Localization of the Legnica–Głogow Copper District (LGCD) on Polands’ map.

The Legnica–Głogow Copper District (LGCD), located in Southwest Poland in Lower Silesian Voivodeship (see Figure 6), is characterized by rock burst phenomena due to the high level of copper exploitation and the geological profile [34,35]. So that, a network of seismic stations has been assembled since the late 1980 s. The recorded accelerations are available and two mining-induced shocks of different spectral characteristics were selected to study the dynamic behavior of the footbridge. Both shocks can be referred to as strong for the LGCD area in terms of both the maximum amplitudes and the energy released.

**Figure 7:**
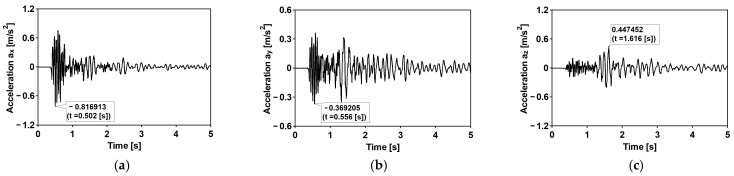
The time history of the registered ground acceleration for the first mining-induced shock: (**a**) horizontal direction WE; (**b**) horizontal direction NS; (**c**) vertical direction Z.

**Figure 8:**
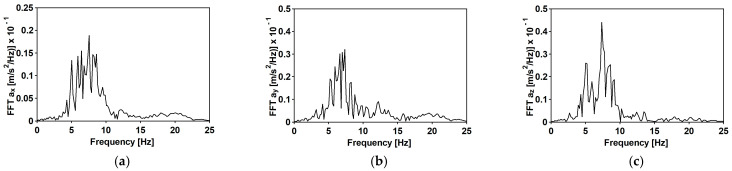
Fourier spectra of the first mining-induced shock: (**a**) horizontal direction WE; (**b**) horizontal direction NS; (**c**) vertical direction Z.

The time histories of accelerations in three directions and the Fourier spectra of the first typical shock [34] are shown in Figure 7 and Figure 8, respectively. The duration of the intense vibration phase was about 2.5 s. The peak ground acceleration (PGA) levels reached 0.81 and 0.39 m/s^2^ for the west–east (WE) and the north-south (NE) directions, respectively. The maximum vertical component acceleration did not exceed 0.45 m/s^2^. The spectral analysis of the shock revealed that the dominant frequencies fall into quite a narrow and compact frequency range 5–10 Hz. 

**Figure 9:**
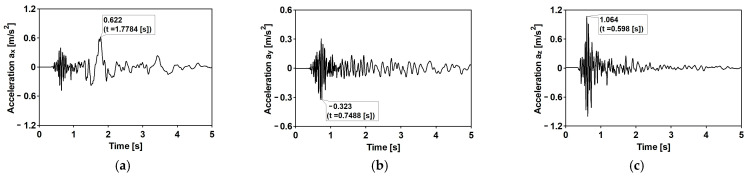
The time history of the registered ground acceleration for the second mining-induced shock: (**a**) horizontal direction WE; (**b**) horizontal direction NS; (**c**) vertical direction Z.

**Figure 10:**
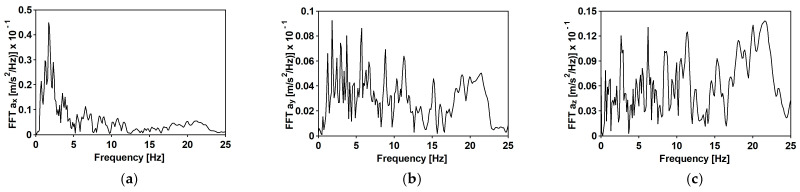
Fourier spectra of the second mining-induced shock: (**a**) horizontal direction WE; (**b**) horizontal direction NS; (**c**) vertical direction Z.

The second mining-induced shock, also recorded by a local seismic station in the LGCD [22], has different spectral characteristics. The duration of the intense vibration phase was about 5.50 s. The maximum PGA levels reached 0.62 and 0.32 m/s^2^ for the west–east (WE) and the north–south (NE) directions, respectively. The maximum vertical component acceleration did not exceed 1.20 m/s^2^. The time histories of accelerations in three directions and the Fourier spectra are shown in Figure 9: differently than the first event (Figure 8), the shocks (Figure 10) are scattered up to 20 Hz in NS and Z directions.

## 4. Adopted Finite Element Model for Numerical Analyses

**Figure 11:**
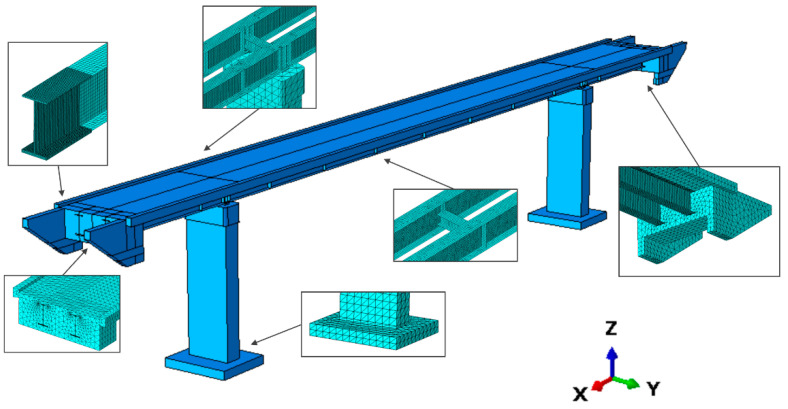
The numerical model of the footbridge with elements and mesh details.

The finite element (FE) elastic model of the footbridge (Figure 11) was implemented in ABAQUS/Standard [36]. The 8-node and 3-node brick finite elements were used to model concrete elements and transverse steel beams, and the steel beams of the girders were modeled by 3-node shell elements. The total number of elements was about 316,000, and the element dimensions were determined based on the convergence analysis, with the first natural frequency value serving as the convergence criterion. Kinematic coupling constraints are given between the bottom of the girders and the top of the pillars based on the different bearing types applied in the structure. Due to efficiency reasons, non-structural elements, such as barriers, even if considered as masses were not considered as structural elements, having negligible influence on the dynamic response.

Fixed boundary conditions, reflecting the high rigidity of the foundations as well as the high stiffness of the subsoils, were applied at the ends of the piers. 

**Figure 12:**
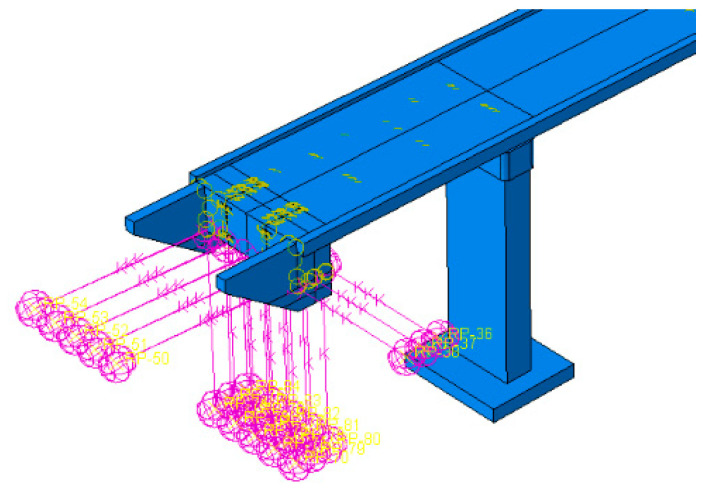
Boundary conditions for the SPRING_Ovhg.

As far as the SPRING_Ovhg is concerned (Figure 3c), the equivalent strings position is reported in Figure 12: for both bridge end parts, a set of 15 springs has been considered, placing them either on three (longitudinal and vertical directions) or five planes (transversal direction). 

## 5. Soil Characterization vs. Springs’ Constants Estimation

Numerical analyses that take into account DSSI are based on the 3D models reported in Figure 11 and Figure 12 that include linear springs whose dynamic stiffnesses $C_{Z}$, $C_{X/Y}$ for a unitary area, have been evaluated based on the Savinov method [8,37,38]. The Savinov method, commonly applied in Poland, gives, as documented in [8], stiffness values in agreement with those predictable through the expressions reported in ASCE 4-98 [39] and SP 26.13330.2012 [40] standards. The Savinovs’ Equations (Equations (1) and (2)), holding if the contact area is smaller than 50 m^2^, depend on (1) the trial coefficient $C_{0}$ evaluated as reported in Table 2; (2) the on-ground static pressure in MPa; (3) the dimensional coefficient $\Delta_{1}=1.0\;m^{-1}$; (4) the contact area dimensions (*B*, *L*).

(1) $$C_{Z}=C_{0}\left[1+\frac{2\left(B+L\right)}{\Delta_{1}\cdot \left(B\cdot L\right)}\right]\sqrt{\frac{p}{p_{0}}},$$

(2) $$C_{X/Y}=0.70C_{z}.$$

Having calculated the coefficients $C_{Z}$, *C_X/Y_* (Equations (1) and (2), the global dynamic soil stiffness can be evaluated (Equations (3) and (4)) as function of the contact area (*A*):

(3) $$k_{Z}=C_{Z}\cdot A,$$

(4) $$k_{X/Y}=C_{X/Y}\cdot A.$$

The dynamic soil stiffness for vertical ground ($k_{Z})\;$and horizontal ground ($k_{X/Y})$ base motion can be interpreted as the constants of concentrated springs attached at the ends of the overhangs in the vertical and horizontal directions, respectively. Considering, in each direction, *n* = 15 springs, the stiffness ($k_{i}$) of each spring was evaluated, as reported in Equations (5) and (6):

(5) $$k_{i\_Z}=\frac{k_{Z}}{n},$$

(6) $$k_{i\_X/Y}=\frac{k_{X/Y}}{n}.$$

## 6. Results and Discussion

The assessment of the structure subjected to mining-induced shocks will be discussed in the following sections, in terms of modal models and dynamic behavior. Two seismic scenarios have been considered: trends were recognized and analyzed referring to two structural models which differ for the assumed overhang boundary conditions.

### 6.1. Experimental Identification of Modal Parameters of the Footbridge

**Figure 13:**
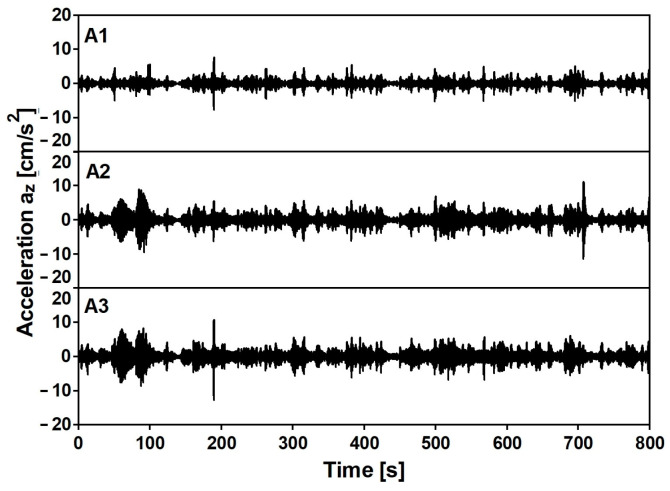
Acceleration [cm/s^2^] time histories at A1, A2 and A3 stations for vertical direction.

The experimental evaluation of modal parameters of the footbridge was conducted using the Operational Modal Analysis (OMA) techniques [26,27,28,29,30]. Modal identification of the footbridge was realized based on the auto- and cross-correlation function of output-only data collected under in-operation field tests. Signals of registered vibrations generated by chaotic pedestrian movement, heavy road traffic under the footbridge, and wind were processed. The length of the recorded signals was 15 min. Examples of recorded time histories are reported in Figure 13.

**Figure 14:**
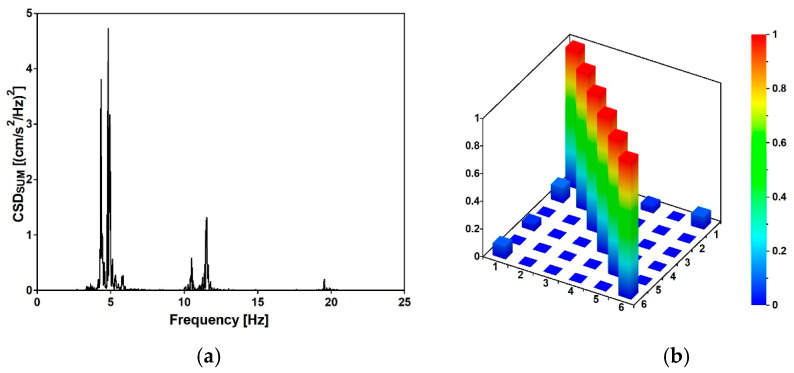
(**a**) Natural frequency estimator: CSD values vs. frequency value; (**b**) 3D visualization the AutoMAC values.

**Figure 15:**
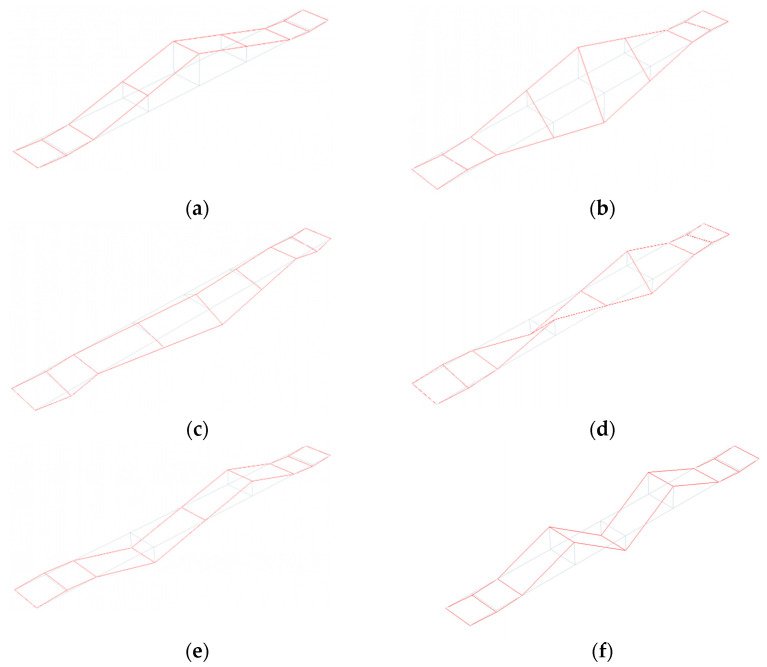
The six experimental mode shapes of the footbridge: (**a**) first (vertical), (**b**) second (torsional), (**c**) third (lateral), (**d**) fourth (vertical), (**e**) fifth (torsional), (**f**) sixth (vertical).

Experimental values of natural frequencies, logarithmic decrements, and damping values are reported in Table 3. The frequencies were obtained on the basis of the estimator, which is the summation of all combinations of cross-spectral density (CSD) functions [27] between the output recorded at each station: the peak picking method [27,30] was used to select the frequencies having the higher CSD values (see Figure 14a). Once the eigenfrequencies were estimated, the Time Domain Decomposition (TDD) method [30] was implemented to determine the corresponding modes, which schematic shapes are reported in Figure 15. Those modes were verified through the Modal Assurance Criterion (MAC) [31], commonly known as AutoMAC, that serves to check the sufficiency of the stations and degrees of freedom. The AutoMAC (see Figure 14b) verification is satisfactory since non-diagonal values are less than 0.2. Hence, the experimental modal model is positively verified in terms of eigenpairs of the modal system [31]. Damping values have been evaluated, during free vibrations, based on logarithmic decrement estimations. At this study stage, the band-pass filter was applied [33] to assess damping properties corresponding to estimated eigenvalues and mode shapes. It should be emphasized that the experimentally detected modal parameters are in good agreement with the data published by other authors for footbridges of similar dimensions and construction details [41,42]. 

**Figure 16:**
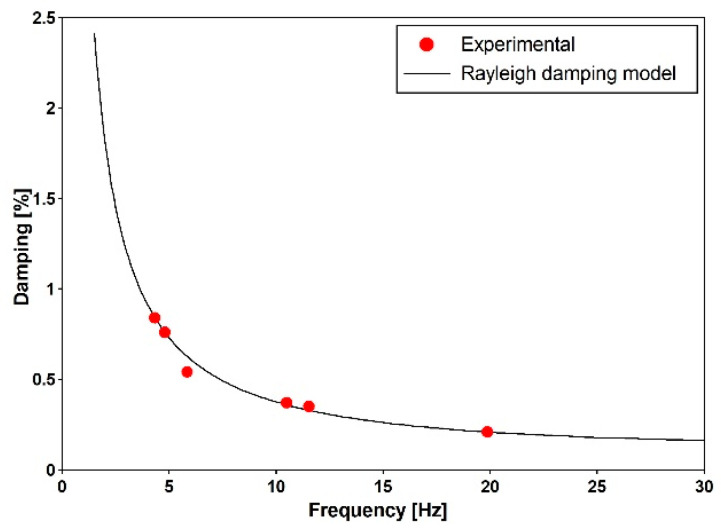
The dependence of the damping ratio on the frequency for the determined Rayleigh coefficients along with the damping ratios detected experimentally for particular mode shapes.

The Rayleigh mass and stiffness proportional damping model was used for the numerical simulations [32]. Based on obtained logarithmic decrements (see Table 3) for the first and fourth modes (both vertical), the coefficients of the Rayleigh damping model were estimated as 0.4548013 for the mass and 0.0000045 for the stiffness proportional damping. The dependence of the damping ratio on the frequency for the determined Rayleigh coefficients along with the damping ratios detected experimentally are portrayed in Figure 16.

### 6.2. Preliminary Finite Element Analysis and Correlation with Experimental Results

#### 6.2.1. Experimental vs. Numerical Frequencies for the FIX_Ovhg and FREE_Ovhg Models

The natural frequencies and vibration mode shapes for the FIX_Ovhg (Figure 3a) and FREE_Ovhg (Figure 3b) were obtained numerically, through the FE model reported in Figure 11. The values of the first six frequencies are reported in Table 4. Comparing the numerical values with the experimental one, it can be stated that: (1) the FREE_Ovhg hypothesis is unacceptable having a percentage error between ≈30 and 70%; (2) as far as the FIX_Ovhg frequencies are concerned (a) good compliance, with the experimental values, can be argued for the first, second, fourth, and fifth frequencies, where the discrepancies do not exceed 4% making them acceptable values, being lower than 15% [43,44]; (b) the third transversal modes (Table 4) do not acceptable values, having a percentage error of 26.15%. Hence, the goal was to update the FE model to match the numerical frequencies and modal shapes with those evaluated based on the elaboration of the in situ-acquired signals.

#### 6.2.2. Updating the FE Model by Taking the DSSI into Account

Discrepancies between experimental and numerical frequencies could generally be attributed to structural geometry, material properties, and boundary conditions. The structural geometry, as well as the material properties of the analyzed footbridge, were fully recognized in the design and construction stage so that the FE model, denoted as SPRING_Ovhg (Figure 3c), was updated, changing the boundary conditions of the overhangs’ ends, including the set of springs already discussed and reported in Figure 12. The constants of the springs were theoretically evaluated.

The theoretical estimation of the spring constants was evaluated as already discussed in Section 5: (1) due to the soil types, $C_{0}=16\;\mathrm{MPa}/m\;$was assumed; (2) based on the assumed contact areas (Figure 4b,c), the dynamic stiffness of the ground along (a) the vertical direction (Equation (1)) was assumed equal to $k_{Z}^{thr}{}_{\;}=254.96\;\mathrm{MN}/m$, so that each of the 15 assumed springs have a stiffness of $k_{i\_\;Z}^{thr}=17.00\;\mathrm{MN}/m$; (b) the two horizontal directions, having the same contact areas and spring number (n=15), was assumed (Equation (2)) $k_{X/Y}=178.47\;\mathrm{MN}/m\;$so that stiffness of the single springs was $k_{i\_\;X/Y}^{thr}=11.90\;\mathrm{MN}/m$.

**Figure 17:**
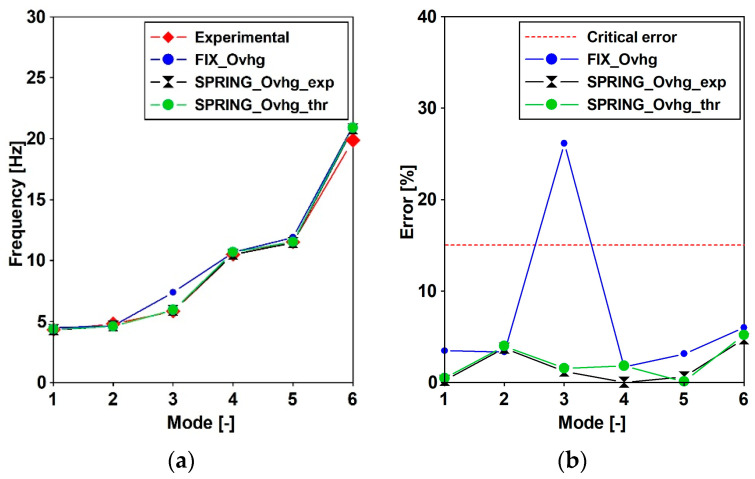
Sensitivity of the natural frequencies vs. adopted model: (**a**) evaluated values [Hz]; (**b**) percentage error.

**Figure 18:**
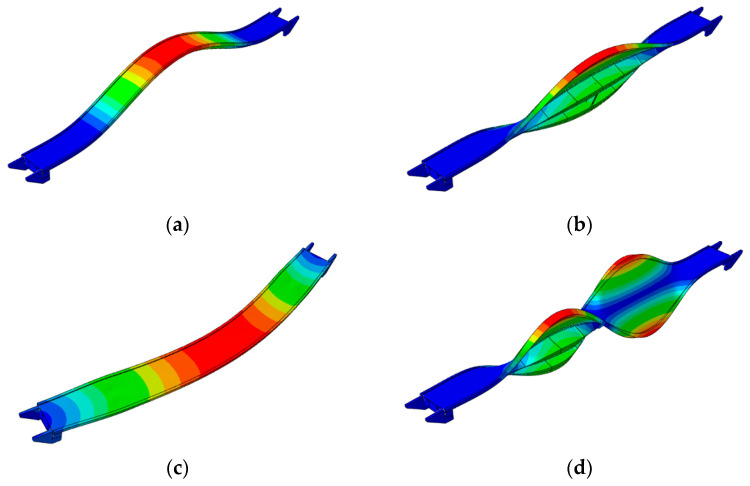

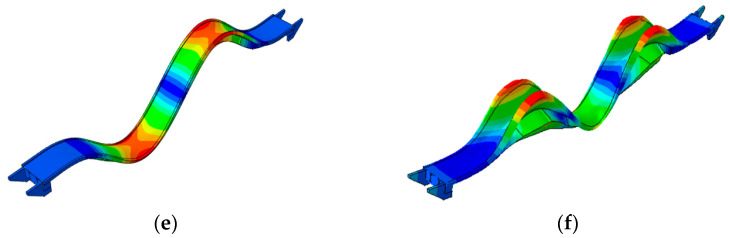
The six basic mode shapes of the footbridge: (**a**) first (vertical), (**b**) second (torsional, (**c**) third (lateral), (**d**) fourth (vertical), (**e**) fifth (torsional), (**f**) sixth (vertical).

**Figure 19:**
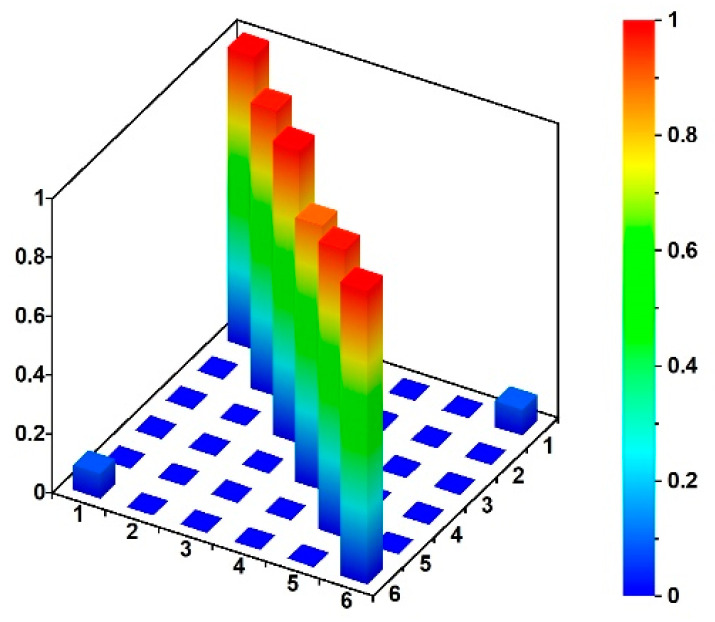
3D visualization of the MAC values showing the correlation between numerical and experimental modal models.

The theoretical evaluations of the spring constant served as support for the trial and error procedure carried out to identify those spring stiffnesses that allow the fitting of frequencies and modal shapes of the numerical model with those obtained through the OMA: (1) the resulting stiffness of the single springs resulted in being equal to $k_{Z}^{num}=224.19\;\mathrm{MN}/m\;$for vertical direction and $k_{\;X/Y}^{num}=156.93\;\mathrm{MN}/m$ for horizontal directions, respectively; (2) the numerically obtained modal shapes as well as the frequencies had an excellent agreements with those experimentally evaluated (Figure 15, Figure 17, Figure 18 and Figure 19 and Table 5).

As far as frequencies are concerned (Table 5), the maximum percentage error resulted equal to 4.73% while in average resulted lower than 2%. However, it has been noticed that, as far as the spring theoretical values are concerned, the fitting can be considered satisfactory as well, having averaged value slightly greater than 2% and a maximum value (5.18%) greater but lower than the threshold of 15%. A summary of the resulting errors is reported in Figure 17 were, for the six considered modes, the experimental frequency is compared in terms of frequency values (Figure 17a) and percentage errors (Figure 17b) that for the FIX_Ovhg model clearly exceed the 15% critical value (red line). 

Regarding modal shapes, those numerically evaluated (see Figure 18) well fit those evaluated through OMA (see Figure 15). For both strategies, their normed eigenvectors, along the alignments A, B (see Figure 5a), for the six considered modes, are reported in Table 6: (1) as far as the third horizontal mode is concerned the horizontal vector components are reported, while (2) regarding the other modes the vertical vector components were considered. The averaged percentage error has been evaluated for each normed eigenvector of the two alignments. The greater error value resulted in close to 5% for mode 3 of alignment A and 6% for mode 3 of alignment B.

The MAC proved a high degree of consistency between numerical and experimental evaluations: the observed boundaries of the MAC values (Figure 19) are greater than 0.80 on the diagonal and less than 0.20 out of the MAC matrix’s diagonal.

### 6.3. Performance of the Footbridge under the Mining-Induced Shocks

The issue of the DSSI is rarely addressed for the analyses of footbridges located in mining areas, especially for the here considered typology. So that the main purpose of the carried out analyses was to compare the dynamic response of the footbridge to mining-induced shocks considering or not considering the DSSI; consequently for both FIX_Ovhg (Figure 3a) and SPRING_Ovhg (see Figure 3c) a comparative study was carried out considering the two mining-induced shocks reported in Figure 7 and Figure 9 of Section 3: the first event had a narrowband spectrum (Figure 8) with frequencies 5–10 Hz, whereas the spectrum (Figure 10) of the second shock was scattered and covered frequencies up to 20 Hz.

**Figure 20:**
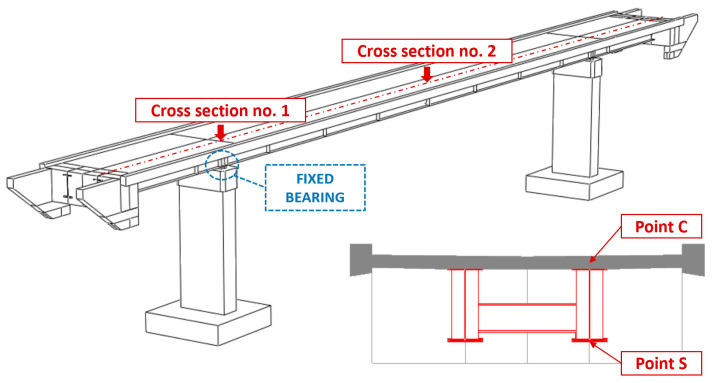
Representative points of the footbridge selected for the dynamic analyses.

The Time History Analysis (THA) was used for dynamic analyses. The calculations were conducted with the Hilber–Hughes–Taylor time integration algorithm provided in the ABAQUS/Standard software [36]. The experimentally estimated Rayleigh damping coefficients were used according to the damping values reported in Table 3 and Figure 16 (Section 6.1). The results, discussed in the following sections, regard the induced stresses at points C and S (Figure 20), respectively, concerning the upper part of the concrete slab and lower part of the bottom flange. Those points regard the two cross sections number 1 and 2, respectively, located over the pillar and at the middle of the central span. Maximal principal stresses $\sigma_{\;}^{princ}$ and von Mises stresses $\sigma_{\;}^{mises}$ were considered, respectively, for concrete slab and steel flange. 

**Figure 21:**
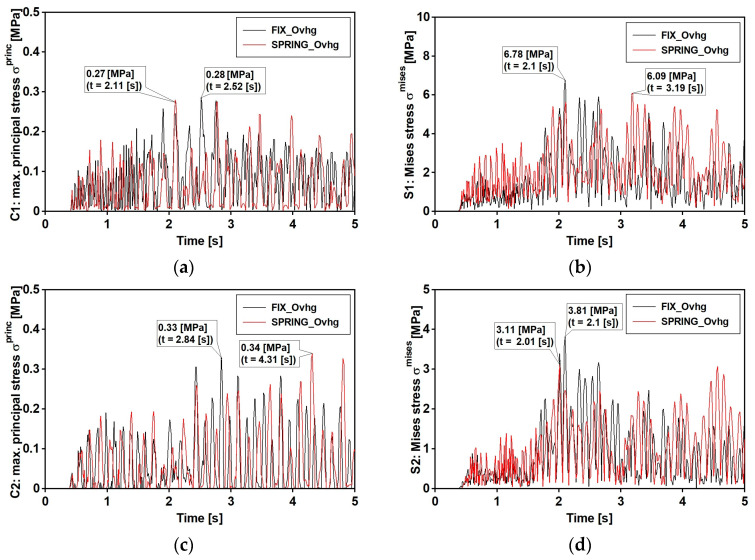
Mining-induced stress [MPa] time histories: event with narrow band spectrum. FIX_Ovhg model (black line) and SPRING_Ovhg model (red line): (**a**) positive value of principal stresses $\sigma_{\;}^{princ}\;$at points C1 (**b**) positive value of principal stresses $\sigma_{\;}^{princ}\;$at points C2; (**c**) the von Mises stresses $\sigma_{\;}^{mises}$ for points S1; (**d**) the von Mises stresses $\sigma_{\;}^{mises}$ for points S2.

**Figure 22:**
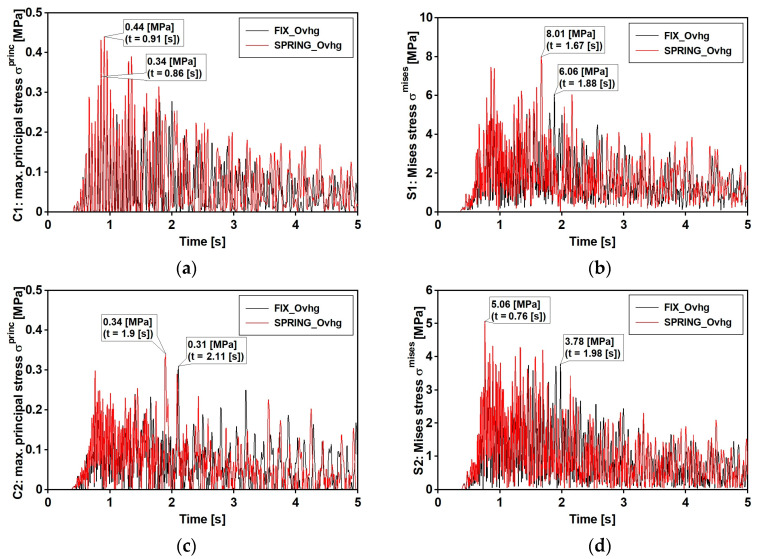
Mining-induced stress [kPa] time histories: event with wide band spectrum. FIX_Ovhg model (black line) and SPRING_Ovhg model (red line): (**a**) positive value of principal stresses $\sigma_{\;}^{princ}\;$at points C1 (**b**) positive value of principal stresses $\sigma_{\;}^{princ}\;$ at points C2; (**c**) the von Mises stresses $\sigma_{\;}^{mises}$ for points S1; (**d**) the von Mises stresses $\sigma_{\;}^{mises}$ for points S2.

**Figure 23:**
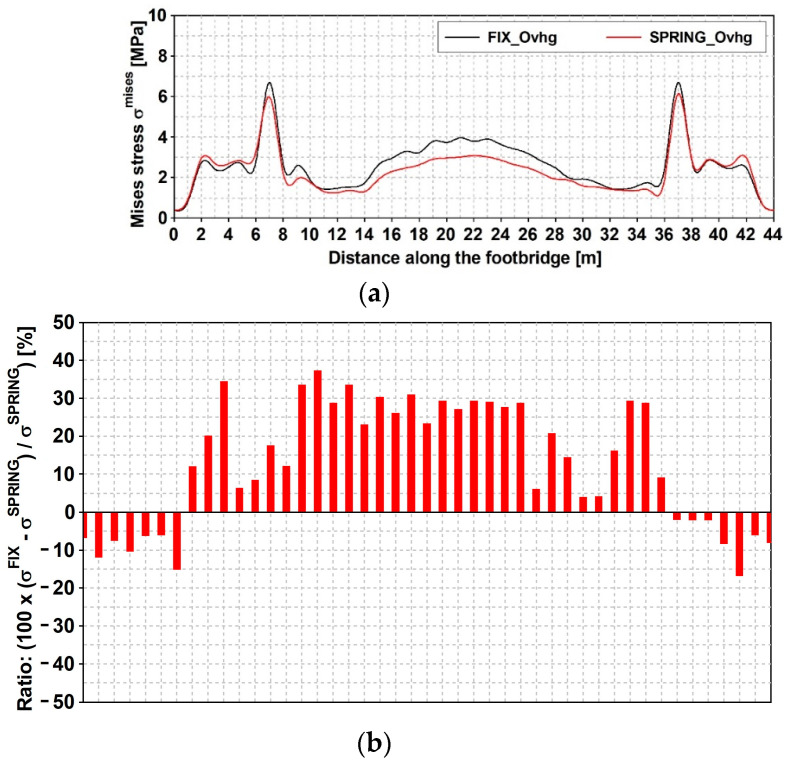
Mining induced stress [MPa] time histories: event with narrow band spectrum; von Mises stress along the span, at the bottom flange: (**a**) FIX_Ovhg (black line) and SPRING_Ovhg model (red line); (**b**) percentage error.

**Figure 24:**
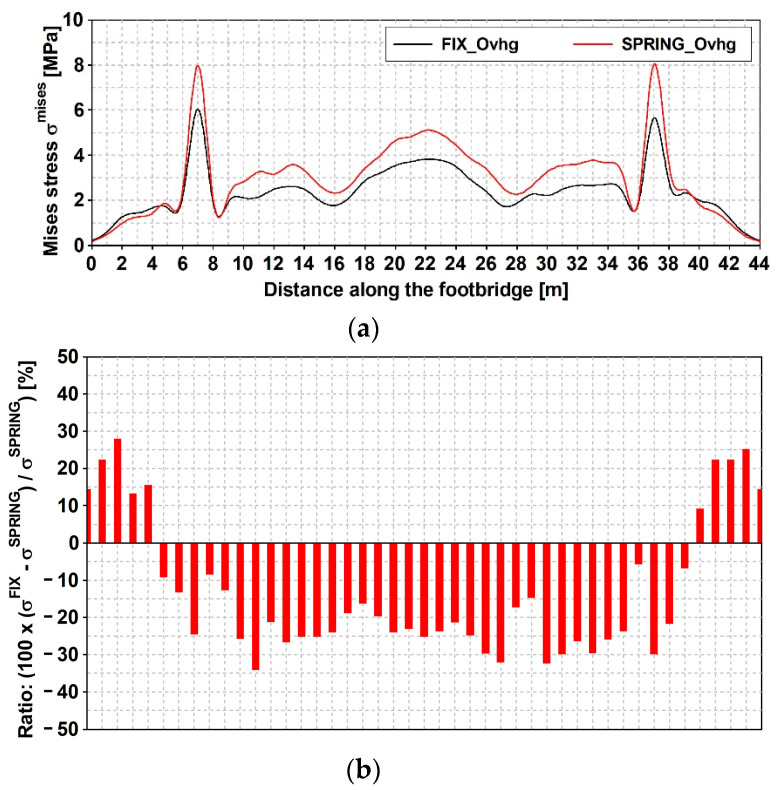
Mining induced stress [MPa] time histories: event with wide band spectrum; von Mises stress along the span, at the bottom flange: (**a**) FIX_Ovhg (black line) and PRING_Ovhg model (red line); (**b**) percentage error.

The time histories of previous defined stress are reported in Figure 21 and Figure 22, respectively, concerning the narrow and wide band event: fix and spring model are considered. Maximum values of those stresses (see Table 7) denote that: (1) as far as that the narrow band shock is concerned, the FIX model denotes greater values than those gained with the SPRING in all examined point with the exception of point C2; (2) different trend concerns the wide band spectrum shock which related stresses are always greater as far as the spring model is concerned; (3) the absolute value of the maximum are in between 23% and 26%. Previous trends can be extended to other sections placed along the span, as outlined by Figure 23 and Figure 24 that concern the narrow and wide band shocks: in both Figures are reported the von Mises stress at the bottom flange (Figure 23a and Figure 24a) of the SPRING and FIX model as well as the percentage error (Figure 23b and Figure 24b) of the FIX model value with respect of the SPRING one.

For the qualification and quantification of the SSI impact on the dynamic response level, the stresses’ ratio (see Figure 23b and Figure 24b) seems to be the best way of the results’ demonstration. It unquestionably portrays how the type of Fourier spectrum of the excitation affects the level of the dynamic response of the footbridge. The response level is up to 40% higher for the FIX_Ovhg model than for the SPRING_Ovhg one as far as the compact spectrum is concerned. The opposite situation can be noticed for the scattered shock spectrum.

Various trends in the DSSI impact on the dynamic response level of the footbridge should be discussed in depth in the context of different spectral characteristics of mining-induced shocks, since it seems to be of crucial importance to the obtained results.

**Figure 25:**
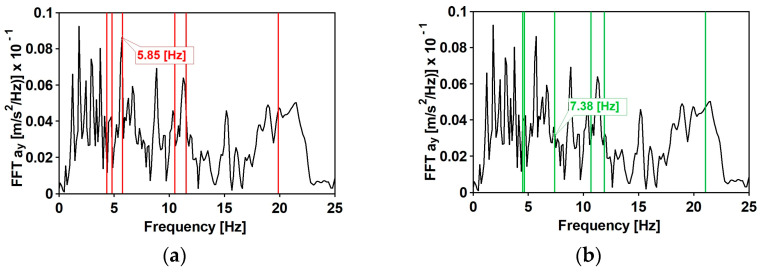
The natural frequencies of the footbridge against the background of the horizontal component of the wide spectrum shock: (**a**) experimentally obtained natural frequencies of the footbridge; (**b**) the natural frequency obtained from FIX_Ovhg model.

As far as the compact frequency spectrum is concerned, results made visible a beneficial effect of the DSSI. While considering the effect of the scattered frequency spectrum, only the transversal component plays a central role: (1) the third horizontal mode has a frequency of 5.85 Hz (see Table 3 and Figure 15), where the spectrum (see Figure 25a) has the higher value; (2) the third lateral mode of the FIX_Ovhg model has a frequency of 7.38 Hz (see Table 5), where a lower spectral value (see Figure 25b) can be observed.

## 7. Conclusions

The impact of the DSSI on the dynamic performance of a single-span concrete-steel composite footbridge with overhangs partially supported on the ground can be of a great importance in the context of mining seismicity, especially in the case of wideband shocks that often occur. Numerical prediction needs to be performed based on FEM models tuned true OMA: frequencies and modes have to include transversal and vertical shapes that are both determinants in the dynamic response of the footbridges. So that it can be stated that:

1. The first six natural frequencies, modes shapes, and damping ratios of the single-span footbridge with overhangs were estimated experimentally. The obtained modal parameters are consistent with the values given in the literature for such structures.
2. The natural frequencies determined numerically for the scheme used usually in static calculations, i.e., a simple supported multi-span beam, turned out to be absolutely inconsistent with the experimental values.
3. The model assuming full restraint of the overhangs’ ends gave the natural frequencies closer to the experimental ones. However, the average error of 7% was also far above expectancy. Especially in the case of the third frequency accompanied by the lateral mode, the error of around 26% was unacceptable, concerning that the maximum tolerable error is 15% [43,44].
4. The adopted strategy for the FEM model tuning included a set of horizontal and vertical elastic springs to consider DSSI between the end block of the overhangs and the ground. The experimentally and theoretically determined spring constants remain in good agreement. The tuned model with the springs has been verified, and good modal compliance was achieved.
5. The impact of the DSSI between the overhangs’ ends on the dynamic response level of the footbridge, in terms of maximal principal stresses for the concrete slab and mises stresses, were evaluated: the DSSI can either amplify or reduce, depending on the spectral characteristics, the mining-induced excitation.
6. In the case of the mining-induced shock with a narrowband spectrum, the stresses, determined including the DSSI, were 40% lower than those determined for the fixed overhangs’ ends. However, in the case of the wideband shock, due to the resonance effects, lateral vibrations take on values high enough to outweigh the reduction in amplitudes usually accompanied by the SSI. It led to a 40% increase in the dynamic response level.

## Figures and Tables

**Table 1:** Material parameters and element masses of the footbridge.

Material Parameters				
Material	Mass Density [kg/m^3^]	Elasticity Modulus [GPa]		Poisson’s Ratio [−]
Concrete	2400.00	37.80		0.30
Structural steel	7850.00	205.00		0.30
**Element masses [ton]**				
Steel beams	Concrete slab	Concrete block	Piers	Non structural part
26.11	72.92	29.75	73.74	5.71

**Table 2:** Savinov method [38]: suggested values for $C_{0}$ [MPa/m].

Dynamic Ground Category	Ground Stiffness Conditions	Soil (Voids Ratio *e*; Consistency Index *I_L_*)	*C*_0_ $\mathrm{at}\;p_{0}=0.02\;[\mathrm{MPa}]$ $\;\mathrm{and}\;F<50.00\;m^{2}\;$
I	Very small stiffness	Clayey sands, dusts, clays and loams in plastic state (*I_L_* = 0.40 ÷ 0.50)	6.00
II	Small Stiffness	Clayey sands, dusts, clays and loams in plastic state (*I_L_* = 0.40 ÷ 0.50)	8.00 ÷ 10.00
		Dusty sands, saturated (*e* > 0.80)	12.00
III	Medium stiffness	Clayey sands, dusts, hard-plastic clays and loams (*I_L_* = 0 ÷ 0.25)	16.00 ÷ 20.00
		Dusty sands, dense and medium density	14.00
		Fine sands, medium sands, coarse sands	18.00
IV	High stiffness	Sandy clays, semi-compact and compact clays, and loams (*I_L_* < 0)	22.00 ÷ 30.00
		Gravel and rubble	26.00

**Table 3:** Experimentally detected natural frequencies and logarithmic decrements of damping.

Mode No.	1	2	3	4	5	6
Experimental Frequency [Hz]	4.34	4.80	5.85	10.50	11.53	19.87
Logarithmic decrement [−]	0.053	0.048	0.034	0.023	0.022	0.013
Critical Damping [%]	0.84	0.76	0.54	0.37	0.35	0.21

**Table 4:** Comparison of the experimental and the numerical values of natural frequencies.

Frequency No.	Experimental Investigation	Numerical Investigation			
		FREE_Ovhg Model		FIX_Ovhg Model	
	Frequency [Hz]	Frequency [Hz]	Error [%]	Frequency [Hz]	Error [%]
1	4.34	2.38	45.16	4.49	3.46
2	4.80	2.93	38.96	4.64	3.33
3	5.85	4.16	28.89	7.38	26.15
4	10.50	4.37	58.38	10.68	1.71
5	11.53	5.08	55.94	11.89	3.12
6	19.87	5.37	72.97	21.06	5.99

**Table 5:** Natural frequencies: theoretical and numerical vs. experimental values for the FIX_Ovhg and SPRING_Ovhg models of the footbridge.

Mode No.	Experimental (OMA) Investigation	Numerical Investigation on the FIX_Ovhg Model		Numerical Investigation on the SPRING_Ovhg Model			
				Experimentally Detected Spring Constants		Theoretically Assumed Spring Constants	
	Frequency [Hz]	Frequency [Hz]	Error [%]	Frequency [Hz]	Error [%]	Frequency [Hz]	Error [%]
1	4.34	4.49	3.46	4.33	0.23	4.36	0.46
2	4.8	4.64	3.33	4.62	3.75	4.61	3.96
3	5.85	7.38	26.15	5.92	1.20	5.94	1.54
4	10.5	10.68	1.71	10.5	0.00	10.69	1.81
5	11.53	11.89	3.12	11.46	0.61	11.52	0.09
6	19.87	21.06	5.99	20.81	4.73	20.90	5.18
Average error [%]			7.29		1.75		2.17

**Table 6:** Mode values evaluate through OMA and Numerical Identification (in parenthesis).

Alignment A	A1	A2	A3	A4	A5	Error %
Mode 1	−0.05(−0.05)	0.66(0.63)	0.94(0.93)	0.66(0.65)	−0.05(−0.05)	1.42
Mode 2	0.01(0.01)	−0.60(−0.61)	−0.93(−0.92)	−0.60(−0.62)	0.01(0.01)	1.22
Mode 3	0.25(0.23)	0.75(0.72)	0.96(0.92)	0.78(0.79)	0.25(0.23)	5.09
Mode 4	−0.07(−0.07)	0.87(0.89)	0.00(0.00)	−0.88(−0.89)	0.07(0.07)	0.69
Mode 5	−0.23(−0.21)	0.90(0.86)	0.00(0.00)	−0.89(−0.86)	0.20(0.19)	4.30
Mode 6	−0.12(−0.13)	0.86(0.87)	−0.62(−0.64)	0.85(0.86)	−0.12(−0.13)	4.45
Error %	4.17	3.02	1.59	1.97	3.56	
Alignment B	B1	B2	B3	B4	B5	Error %
Mode 1	−0.05(−0.05)	0.61(0.62)	0.91(0.93)	0.61(0.63)	−0.05(−0.05)	1.42
Mode 2	0.01(0.01)	0.59(0.59)	0.93(0.94)	0.61(0.63)	0.01(0.01)	0.87
Mode 3	0.20(0.23)	0.70(0.73)	0.85(0.88)	0.75(0.78)	0.23(0.22)	6.23
Mode 4	0.07(0.07)	−0.86(−0.86)	0.00(0.00)	0.86(0.89)	−0.07(−0.07)	0.70
Mode 5	0.22(0.21)	0.86(0.88)	0.00(0.00)	−0.81(−0.88)	0.20(0.19)	4.10
Mode 6	−0.12(−0.13)	0.89(0.87)	−0.62(−0.64)	0.87(0.87)	−0.12(−0.13)	4.43
Error %	4.65	1.75	1.67	3.78	2.95	

**Table 7:** Mining induced stress: maximum values referred to the events with compact and scattered frequency.

Point	Stress Type	Shock with Compact Frequency Spectrum			Shock with Scattered Frequency Spectrum		
		FIX [MPa]	SPRING [MPa]	Error [%]	FIX [MPa]	SPRING [MPa]	Error [%]
C1	Max. principal stress $\sigma_{\;}^{princ}$	0.28	0.27	3.70	0.34	0.44	−22.73
S1	Mises stress $\sigma_{\;}^{mises}$	6.78	6.09	11.33	6.06	8.01	−24.34
C2	Max. principal $\sigma_{\;}^{princ}$	0.33	0.34	−2.94	0.31	0.34	−8.82
S2	Mises stress $\sigma_{\;}^{mises}$	3.81	3.11	22.51	3.78	5.06	−25.30

## Data Availability

Not applicable.
